# The soil *Mycobacterium* sp. promotes health and longevity through different bacteria‐derived molecules in *Caenorhabditis elegans*

**DOI:** 10.1111/acel.14416

**Published:** 2024-11-19

**Authors:** Limeng Liu, Xusheng Hao, Yang Bai, Ye Tian

**Affiliations:** ^1^ State Key Laboratory of Molecular Developmental Biology Institute of Genetics and Developmental Biology, Chinese Academy of Sciences Beijing China; ^2^ University of Chinese Academy of Sciences Beijing China; ^3^ State Key Laboratory of Plant Genomics, CAS‐JIC Centre of Excellence for Plant and Microbial Sciences Institute of Genetics and Developmental Biology, Chinese Academy of Sciences Beijing China; ^4^ Peking‐Tsinghua Center for Life Sciences, College of Life Sciences Peking University Beijing China

**Keywords:** aging, bacterial derivatives, *C. elegans*, longevity, soil bacteria

## Abstract

Commensal bacteria and their derivatives hold significant promise as therapeutic interventions to delay aging. However, with the diverse nature of the soil microbiome and the long lifespan of mammalian models, the exploration of the influence of soil bacteria and bacteria‐derived molecules on host aging remains limited. We conducted a lifespan screening in *Caenorhabditis elegans* using plant root bacterial collection. Our screening identified 8 genera of bacterial isolates capable of extending lifespan, with *Mycobacterium* sp. Root265 exhibits the most pronounced effect on lifespan extension. Biochemical analysis revealed two specific molecules derived from Root265, polysaccharides (PSs) and arabinogalactan peptidoglycan (AGP), responsible for lifespan extension via *daf‐16‐*dependent and ‐independent pathways, respectively. Notably, AGP exhibited a unique ability to enhance protein homeostasis effectively. Moreover, polar lipids originating from Root265 were found to extend lifespan while mitigating age‐related BAS‐1 decline in neurons. Intriguingly, even brief exposures to these bioactive compounds were sufficient to achieve the lifespan‐promoting effects. We found diverse beneficial bacteria and anti‐aging active compounds from soil bacteria. These findings highlight the potential of exploring bacterial derivatives as therapies targeting aging without the constraints associated with direct microbial interventions.

## Graphical abstract

Screening of natural bacterial isolates for lifespan extension identified *Mycobacterium* sp. Root265 promotes longevity and ameliorates age‐related physiological deteriorations. Biochemical analysis revealed that specific molecules derived from Root265, including polysaccharides (PSs), arabinogalactan peptidoglycan (AGP), and polar lipids, synergistically contribute to anti‐aging effects observed in *C. elegans*.

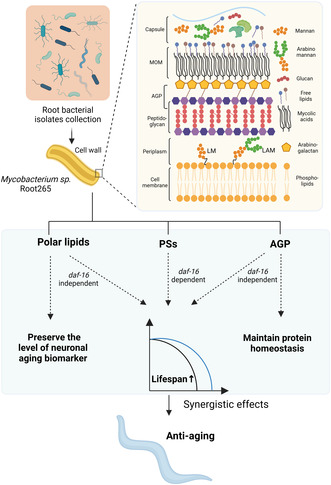

**Abbreviations**
AGP: Arabinogalactan peptidoglycan
C. elegans: Caenorhabditis elegans
FMT: fecal microbiota transplantation
GPLs: glycopepidolipids
mAG: mycolyl‐Arabinogalactan
PG: peptidoglycan
PSs: polysaccharides
SPE: solid phase extraction

## 1 INTRODUCTION

Soil bacteria are usually considered as the provider of pathogen and beneficial microorganisms and have influences on human health, either directly or indirectly (Banerjee & van der Heijden, 2023; Sun et al., 2020). Despite the recognition of soil bacteria as reservoirs of genetic material and active compounds (Crits‐Christoph et al., 2018), the exploration of environmental bacteria's effects on host longevity and the associated functional molecules remains relatively limited.

The abundant and varied microbial communities within the human gastrointestinal tract play a crucial role in influencing both overall health and aging (DeJong et al., 2020; López‐Otín et al., 2023). The utilization of fecal microbiota transplantation (FMT) and probiotic supplementation has gained traction in disease therapy in human (Baruch et al., 2021; Davar et al., 2021; Drekonja et al., 2015; Helmink et al., 2019; Ting et al., 2022) and also prolonged lifespan in mice (Bárcena et al., 2019; Parker et al., 2022). Given the challenges regarding probiotic viability, localization, and potential drawbacks, microbial‐derived molecules have emerged as promising therapeutic alternatives addressing these limitations (McCarville et al., 2020; Rooks & Garrett, 2016). Unveiling the potential of these microbial‐derived molecules and their mechanisms affecting host aging and health represents an intriguing yet complex task. With the intricate nature of mammalian gut microbiota and the lengthy lifespans of mammalian models posing hurdles, a comprehensive understanding of the influence of bacteria‐derived molecules on host aging remains elusive.

*Caenorhabditis elegans*, a free‐living soil nematode, naturally subsists on an assortment of soil bacteria in decaying vegetation, offering an excellent platform for investigating anti‐aging microbial resources and the associated microbial‐derived molecules (Backes et al., 2021; Douglas, 2019; Poupet et al., 2020; Shapira, 2017; Zhang et al., 2017). The influence of bacteria on the physiological traits of *C. elegans*, including development, reproduction, resistance, and longevity, has been established (Ermolaeva & Schumacher, 2014; Kissoyan et al., 2019; Kumar et al., 2019; Macneil et al., 2013; Qi & Han, 2018; Zanni et al., 2015). Genetic and chemical methodologies have identified specific *Escherichia coli*‐derived molecules—colanic acid, indole, methylglyoxal, folate, agmatine and non‐coding RNA—as crucial regulators of host aging (Han et al., 2017; Liu et al., 2012; Shin et al., 2020; Sonowal et al., 2017). In the context of commensal bacteria, *Bacillus subtilis* has been found to produce nitric oxide and quorum‐sensing pentapeptide CSF functioning as probiotics that extend the lifespan of *C. elegans* (Donato et al., 2017; Gusarov et al., 2013). Moreover, commensal lactic acid bacteria have demonstrated longevity‐extending effects (Ikeda et al., 2007; Lee et al., 2015; Nakagawa et al., 2016; Zhao et al., 2017).

In this study, we undertook an extensive investigation of bacterial isolates from natural bacteria reservoirs, specifically a collection of plant root‐derived bacteria. Through our screen, we successfully identified eight genera of bacterial isolates that robustly extend the lifespan of *C. elegans*. Of particular interest, *Mycobacterium* sp. Root265, a non‐pathogenic soil bacterium, emerged as a standout candidate for its capacity to promote longevity and ameliorate age‐related physiological deteriorations in *C. elegans*. Subsequent biochemical purification efforts led to the isolation of two key functional molecules within Root265—polysaccharides (PSs) and arabinogalactan peptidoglycan (AGP). These molecules were found to exert their effects through distinct mechanisms, with AGP notably demonstrating a specific capacity to enhance protein homeostasis. Furthermore, another component, polar lipids originating from Root265, was shown to extend lifespan while mitigating age‐related neuronal aging markers. Collectively, these results provide valuable insight into the potential bacteria and the bioproducts they yield, suggesting bacterial derivatives represent a promising alternative to direct microbial interventions for therapeutic strategies aimed at delaying the aging process.

## 2 RESULTS

### 2.1 Diverse soil bacterial species extend the lifespan of *C. elegans*

**FIGURE 1:**
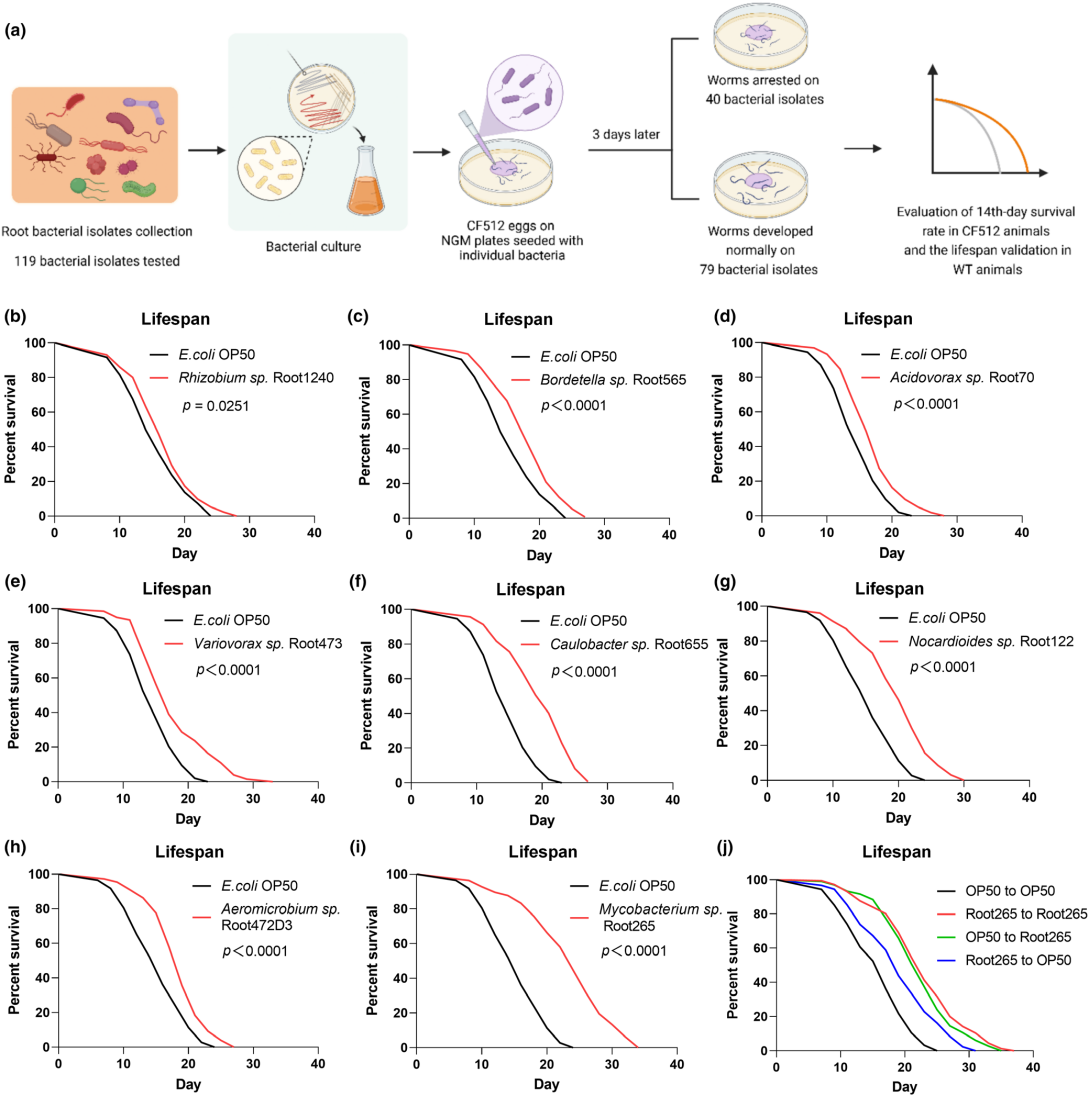
A diverse array of bacterial species extends the lifespan of *C. elegans*. (a), A schematic diagram of bacterial isolates collection screening for lifespan extension in *C. elegans*. Eggs were placed on the plates seeded with individual bacterial cultures to develop into adults. The survival rate was then measured on the same individual bacterial plate to assess the impact of each bacterial isolate on lifespan extension. (b–i), Lifespan analysis of wild‐type (WT) animals fed with different bacterial isolates throughout their entire lifespan, *Rhizobium* sp. Root1240 (b), *Bordetella* sp. Root565 (c), *Acidovorax* sp. Root70 (d), *Variovorax* sp. Root473 (e), *Caulobacter* sp. Root655 (f), *Nocardioides* sp. Root122 (g), *Aeromicobium* sp. Root472D3 (h), *Mycobacterium* sp. Root265 (i). (j), Lifespan analysis of WT worms fed with Root265 during different life stages: Only during the larval stage (Root265 to OP50), only during adulthood (OP50 to Root265), and throughout their entire lifespan (Root265 to Root265).

To explore the impact of soil bacteria on the longevity of *C. elegans*, we conducted a screening of the collection of *Arabidopsis* root bacterial isolates (Bai et al., 2015). This collection encompasses 119 distinct bacterial isolates belong to 39 genera, spanning four prominent phyla: *Proteobacteria*, *Actinobacteria*, *Bacteroidetes*, and *Firmicutes*. Phylogenetic tree was constructed based on nearly full 16S rRNA gene sequences of 105 bacterial isolates (Figure S1a). To minimize worm transfer during the reproductive phase, we employed the sterile worm strain CF512 (*rrf‐3*(*b26*); *fem‐1*(*hc17*)) for the primary lifespan screening. Our assessment focused on the 14th‐day survival ratio, which closely approximated the median survival observed when worms were fed the standard laboratory *E. coli* OP50 (Figure 1a). Among the 119 bacterial isolates tested, 40 were observed to impede the growth of *C. elegans*. Notably, among the remaining isolates, 21 isolates from 9 genera exhibited a significant increase in the 14th‐day survival rate compared to OP50 (Figure S2g and Table S1).

Subsequently, we replicated the lifespan experiments using wild‐type N2 (WT) worms. Our findings consistently demonstrated that bacterial isolates classified under the genera *Nocardioides, Aeromicrobium, Mycobacterium, Rhizobium, Acidovorax, Variovorax, Caulobacter*, and *Bordetella* extended the lifespan of WT *C. elegans*, except for *Pseudomonas* (Figure 1b–1i and Figure S2a–c). This comprehensive analysis establishes that a wide spectrum of bacterial species possesses the capacity to extend the lifespan of *C. elegans*.

### 2.2 *Mycobacterium* sp. Root265 enhances lifespan and mitigates age‐related physiological decline in *C. elegans*

*Mycobacterium* sp. Root265 has emerged as a potent candidate for extending the lifespan of *C. elegans*. Notably, Root265 did not significantly affect the pumping rate (Figure S7a) and was effective in promoting lifespan extension when administered either solely during the larval stage or adulthood (Figure 1j). Additionally, the worms exhibited similar food clearance rates on both OP50 and Root265 (Figure S7e), with no notable difference in calorie intake between two bacteria (Figure S7d). Phylogenetic analysis of the 16S rRNA gene sequence spanning 1436 bp revealed that Root265 shares 99.3% similarity with its closest relative, *M. frederiksbergense* DSM 44346^T^, placing Root265 in a subgroup of rapidly growing, non‐pathogenic *Mycobacterium*, which aligns with its observed growth rate (Figure S1b,S,2f) (Willumsen et al., 2001). We further investigated the effects of other non‐pathogenic Mycobacterial species on the lifespan of *C. elegans*, including *M. smegmatis*, *M. litorale*, and *M. phlei*. Intriguingly, while *M. smegmatis* and *M. litorale* extended *C. elegans* lifespan, *M. phlei* did not (Figure S2d,e), indicating that lifespan modulation may be a shared feature among certain mycobacteria species.

**FIGURE 2:**
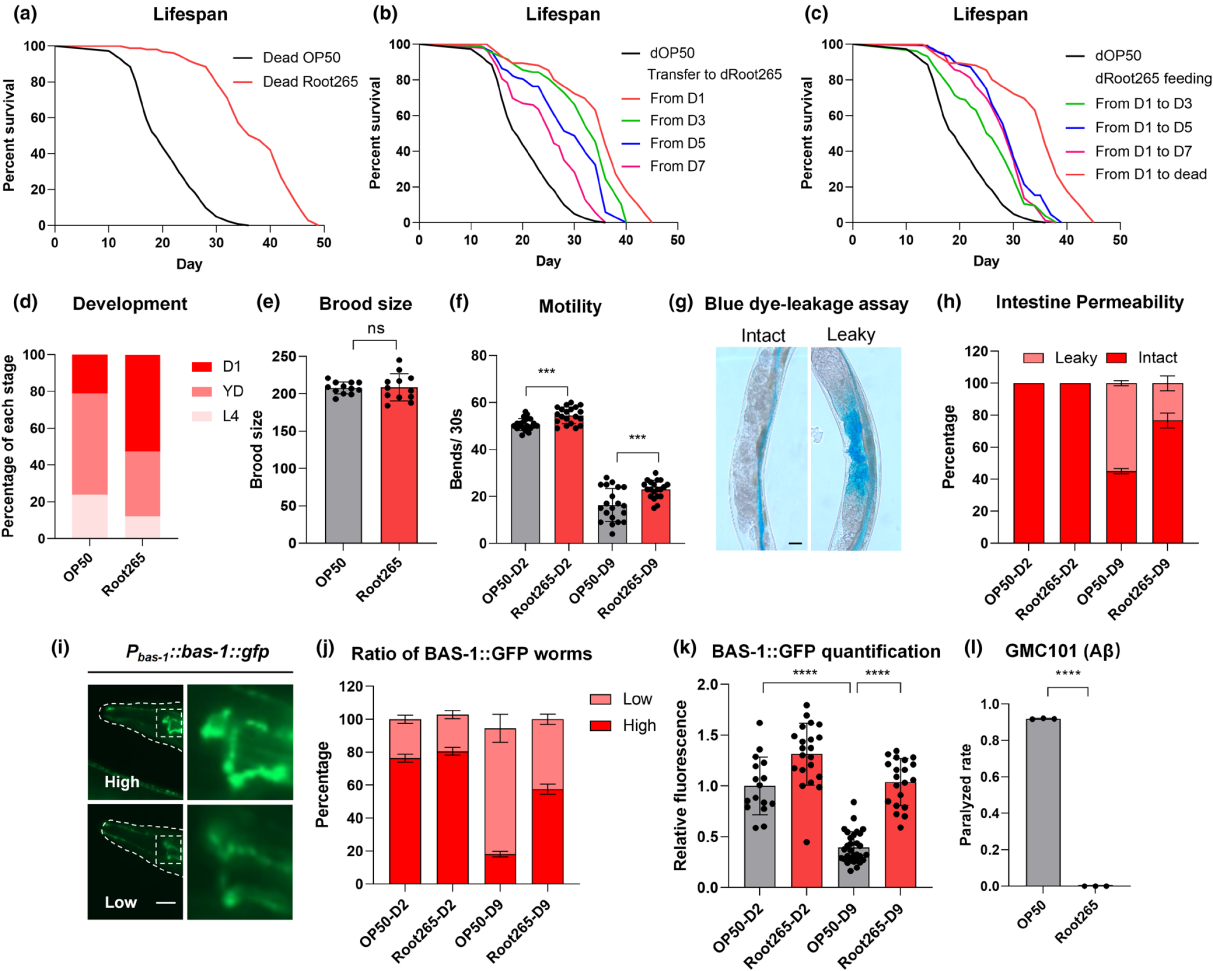
*Mycobacterium* sp. Root265 extends lifespan and ameliorates age‐related physiological deterioration in *C. elegans*. (a) Lifespan analysis of WT animals fed with dead OP50 and dead Root265. (b) Lifespan analysis of WT animals transferred from dead OP50 diet to dead Root265 diet at different stages of adulthood. (c) Lifespan analysis of WT animals with short‐term exposure to dead Root265 during adulthood, followed by a transfer to dead OP50. (d) Developmental rates of animals fed with Root265 and OP50. (e) Brood size of animals fed with Root265 and OP50. *n* = 12 worms. (f) Motility analysis of animals fed with Root265 and OP50 on day 2 and day 9 of adulthood. *n* = 20 worms. (g) Representative images of animals with intact intestines and leaky intestines after soaking in blue food dye. Scale bar, 20 μm. (h) Percentage of animals fed with Root265 and OP50 showing intact and leaky intestines on day 2 and day 9 of adulthood. *n* = 84 (OP50) and *n* = 81 (Root265) for day 2 adult worms, *n* = 157 (OP50) and *n* = 181 (Root265) for day 9 adult worms. (i) Representative fluorescent images of BAS‐1::GFP in animals with high and low expression levels. Scale bar, 20 μm. (j) Percentage of animals fed with Root265 and OP50 with high and low BAS‐1 levels on day 2 and day 9 of adulthood. *n* = 72 (OP50) and *n* = 77 (Root265) for day 2 adult worms, *n* = 100 (OP50) and *n* = 97 (Root265) for day 9 adult worms. (k) Quantitative analysis of BAS‐1 levels performed by measuring GFP fluorescence intensity in the soma of NSM neurons. *n* = 16 (OP50) and *n* = 21 (Root265) for day 2 adult worms, *n* = 30 (OP50) and *n* = 20 (Root265) for day 9 adult worms. (l) Paralyzed rate of GMC101 animals fed with Root265 and OP50. *n* = 3 independent experiments. ****p < 0.0001, ***p < 0.001, ns denotes *p* > 0.05 via unpaired two‐tailed Student's *t* test. Error bars represent SEM. Source data for statistics are provided.

Considering that *C. elegans* typically feed on live bacteria in lab settings, concerns arose regarding potential pathogenicity as these bacteria colonize the intestines of aging worms (McGee et al., 2011). To decipher whether Root265's impact on longevity resulted from altered colonization abilities or metabolites dependent on live bacteria, we employed kanamycin‐killed bacteria as food. Remarkably, worms fed with deceased Root265 still exhibited an extended lifespan (Figure 2a).

The temporal dynamics of Root265's influence on aging were further examined. Lifespan experiments with worms transitioned to kanamycin‐killed Root265 plates at various adult ages revealed a significant extension, even when such transfers occurred as late as Day 7, albeit with reduced effect as aging (Figure 2b). To further investigate the temporal window for Root265's impact on longevity, we implemented a brief exposure regimen, feeding worms with Root265 for only a few days. Impressively, worms exposed to deceased Root265 for just 2 days from the onset of adulthood displayed an extended lifespan (Figure 2c). However, the magnitude of this extension was slightly reduced compared to worms fed Root265 throughout their lifespans. Strikingly, Root265‐fed worms exhibited accelerated maturation compared to those fed OP50 (Figure 2d), while their brood size remained comparable to OP50‐fed worms (Figure 2e). These results uncover the multifaceted potential of *Mycobacterium* sp. Root265 in promoting longevity and shed light on the intricate interplay between Root265 and various aspects of *C. elegans* physiology.

To explore its effects on age‐associated declines in aged worms, we conducted evaluations spanning motility, intestinal barrier integrity, and neuronal functions. Notably, Root265‐fed worms displayed significantly improved motility compared to those fed OP50, particularly on Day 2 and Day 9, indicating a partial delay in age‐related motility decline (Figure 2f).

Age‐related integrity loss in the intestinal barrier is a well‐known phenomenon, leading to heightened intestinal permeability and increased age‐linked mortality risks (Salazar et al., 2023). To assess intestinal integrity in *C. elegans*, we employed a blue dye‐leakage assay (Gelino et al., 2016). As expected, aged worms (Day 9) fed on OP50 exhibited the “Smurf” phenotype, indicative of dye presence in their body cavities. In contrast, fewer “Smurf” worms were observed in those fed Root265, signifying enhanced intestinal barrier integrity (Figure 2g,h).

The decline in dopamine and 5‐HT production with age serves as a biomarker for neuronal aging. Using transgenic worms expressing *Pbas‐1::bas‐1::gfp*—an enzyme central to dopamine and 5‐HT synthesis—we observed a significant reduction in Day 9 worms fed on OP50 (Yin et al., 2014, 2017; Yuan et al., 2020). Remarkably, worms fed Root265 exhibited a notable increase in the proportion of worms with higher BAS‐1 levels (Figure 2i–k), suggesting preserved neurosensory functions.

Additionally, in a transgenic *C. elegans* strain modeling Alzheimer's disease (AD) with toxic full‐length human A‐β_1‐42_ peptide in muscle cells (Mccoll et al., 2012), Root265 was found to contribute to maintaining protein homeostasis and reducing paralysis associated with AD (Figure 2l). These findings suggest that *Mycobacterium* sp. Root265 not only extends worm lifespan but also enhances overall health, highlighting its multifaceted beneficial effects on various physiological aspects of *C. elegans* aging.

### 2.3 *Mycobacterium* sp. Root265 extends lifespan through DAF‐16 and SKN‐1 pathways

**FIGURE 3:**
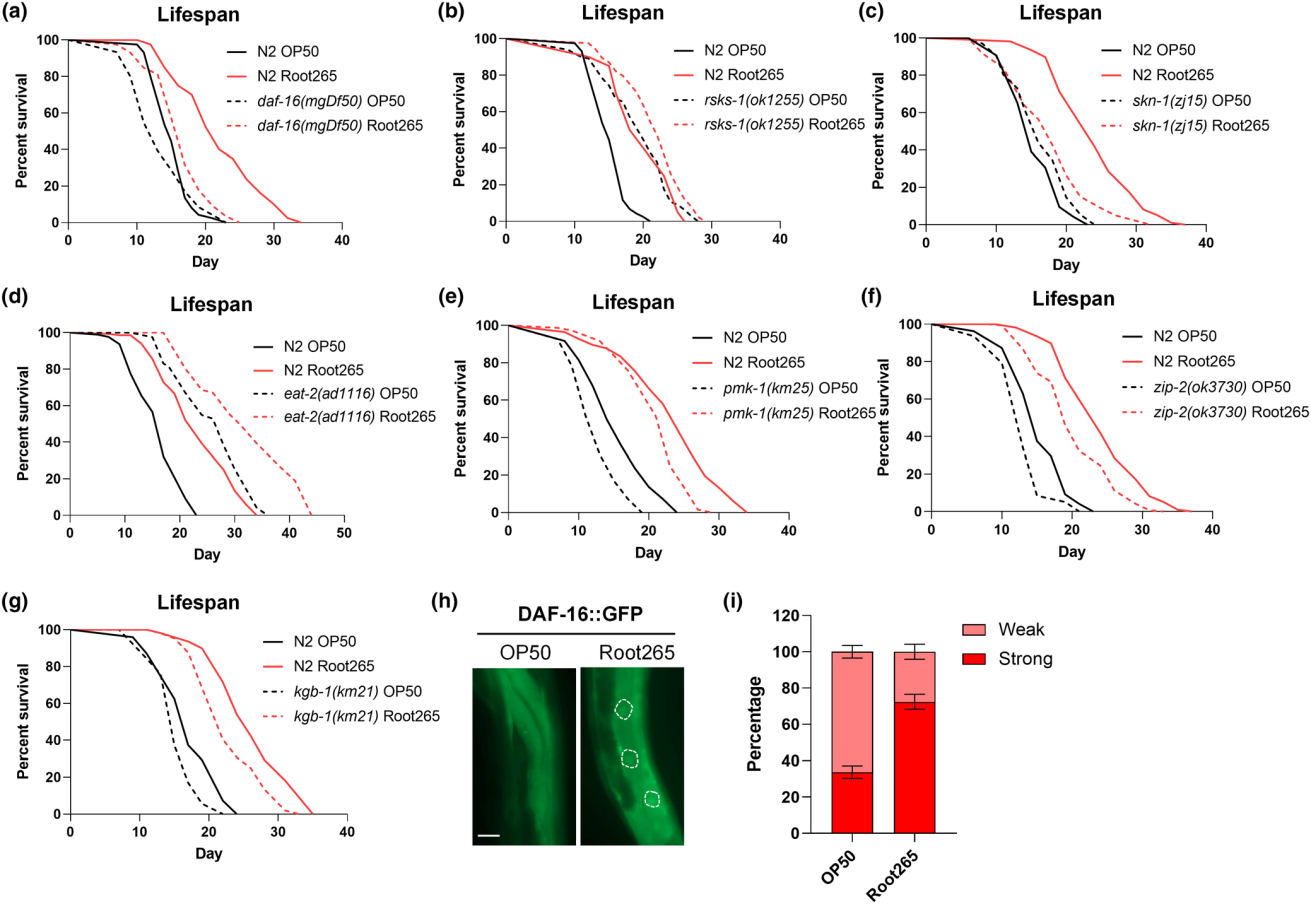
*Mycobacterium* sp. Root265 induces lifespan extension dependent on multiple signaling pathways. (a–g) Lifespan analysis of animals fed with Root265 and OP50 in different genetic backgrounds: (a) *daf‐16*(*mgDf50*), (b) *rsks‐1*(*ok1255*), (c) *skn‐1*(*zj15*), (d) *eat‐2*(*ad1116*), (e) *pmk‐1*(*km25*), (f) *zip‐2*(*ok3730*) and (g) *kgb‐1*(*km21*). (h) Fluorescent image showing DAF‐16 nuclear localization in the intestine on day 4 of adulthood. Scale bar: 20 μm. (i) Percentage of animals fed with Root265 and OP50 exhibiting strong and weak DAF‐16 nuclear localization signals on day 4 of adulthood. *n* = 90 (OP50) and *n* = 85 (Root265).

Our exploration into the mechanisms underlying Root265‐induced lifespan extension led us to examine established signaling pathways governing host longevity (Lapierre & Hansen, 2012). Notably, we observed the abrogation of lifespan extension in Root265‐fed *daf‐16*(*mgDf50*) mutants (Figure 3a). Furthermore, analysis of adult day 4 worms, fed Root265 from day 1 of adulthood, revealed an elevated ratio of DAF‐16 nuclear accumulation (Figure 3h,i). Interestingly, the longevity mutant *daf‐2*(*e1370*) and its downstream kinase AKT‐1 remained responsive to Root265‐induced lifespan extension, indicating that the Root265‐mediated longevity is not through the insulin‐like DAF‐2 signaling pathway (Figure S3a,b).

Since inhibition of the ribosomal S6 kinase RSKS‐1, a central component of the TOR pathway is known to promote lifespan extension (Pan et al., 2007), we explored the impact of Root265 on *rsks‐1*(*ok1255*) mutants. We found that the extent of Root265‐induced lifespan extension was partially diminished in *rsks‐1* mutants (Figure 3b), suggesting partial involvement of the TOR signaling pathway in Root265‐related lifespan modulation.

Considering SKN‐1/Nrf's role in oxidative stress and longevity mediation (Oliveira et al., 2009), we also observed that Root265‐induced lifespan extension was abolished in the *skn‐1*(*zj15*) mutants (Figure 3c) despite the absence of noteworthy transcriptional induction of the glutathione s‐transferase GST‐4 (Figure S3c). Moreover, the extension of lifespan by Root265 persisted in *eat‐2*(*ad1116*) mutants, a genetic model of dietary restriction (DR), and *pmk‐1*(*km25*), *zip‐2*(*ok3730*), and *kgb‐1*(*km21*) mutants lacking innate immune signaling activation (Figure 3d–g). This implies that these pathways are not required for Root265‐induced longevity. In conclusion, our results shed light on the intricate landscape of multiple signaling pathways influencing Root265‐induced longevity, underscoring the multifaceted mechanisms driving bacteria‐mediated extension of lifespan in *C. elegans*.

### 2.4 *Mycobacterium* sp. Root265‐derived polysaccharides prolong lifespan

The extension of lifespan was evident even with kanamycin‐killed and heat‐killed Root265 supplementation (Figure S4a,b), indicating that the whole‐cell components play a role in delaying aging. Given the intricate and distinct cell envelope composition of *Actinomycetota*, particularly mycobacteria, which includes complex lipids, lipoglycans, and glycans (Jankute et al., 2015), we embarked on an investigation into the mechanisms of Root265's longevity benefits.

**FIGURE 4:**
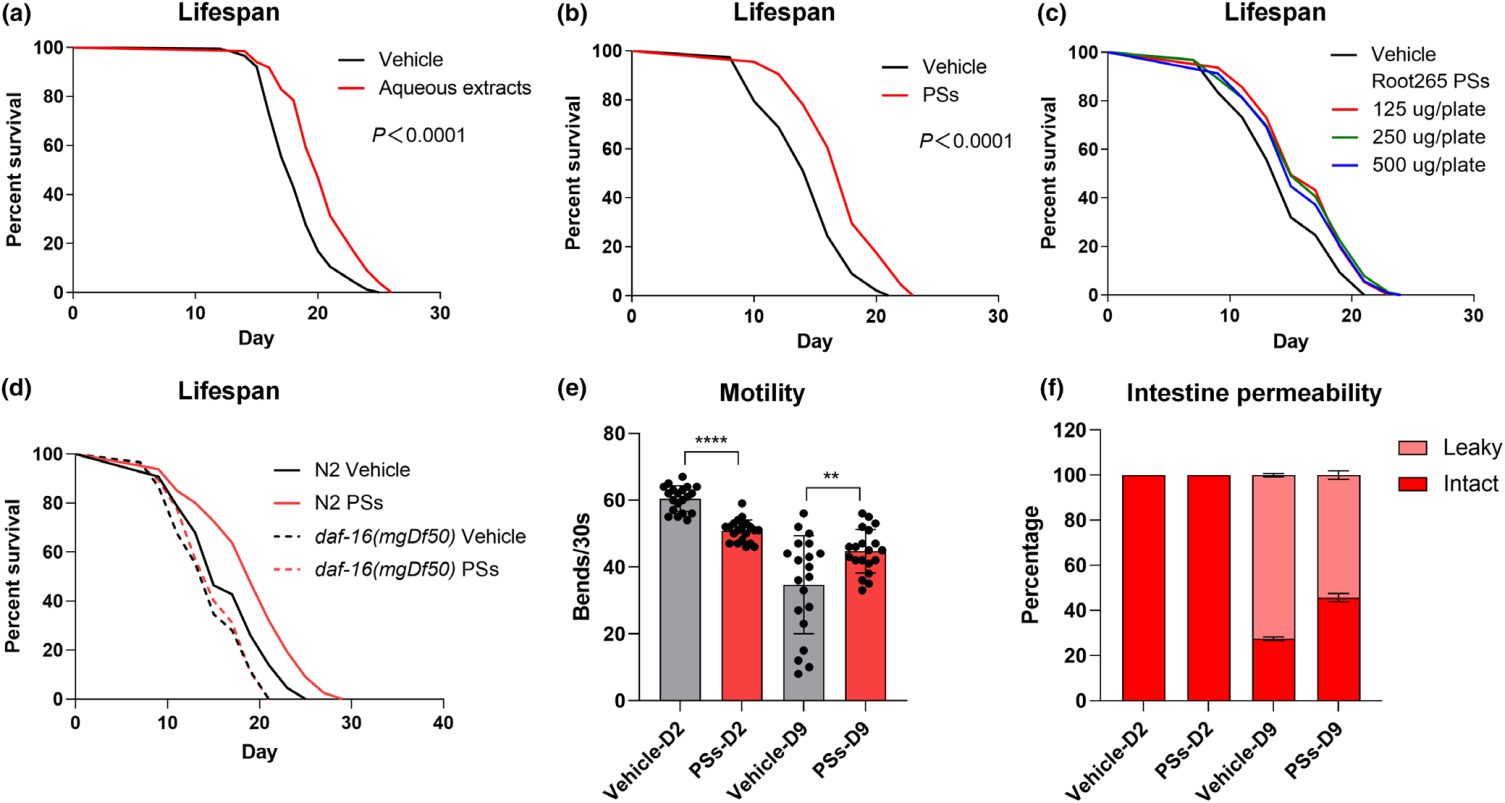
*Mycobacterium* sp. Root265‐derived polysaccharides (PSs) extend lifespan and alleviate age‐related deterioration in muscle and intestinal function. (a, b) Lifespan analysis of WT animals supplemented with aqueous extracts (a) and purified PSs (b) extracted from Root265 during adulthood. (c) Lifespan analysis of WT animals supplemented with different concentrations of Root265‐derived PSs during adulthood. (d) Lifespan analysis of WT and *daf‐16*(*mgDf50*) mutant animals supplemented with Root265‐derived PSs. (e) Motility analysis of animals supplemented with Root265‐derived PSs on day 2 and day 9 of adulthood. *n* = 20 worms. (f), Percentage of animals with intact and leaky intestines on day 2 and day 9 of adulthood, supplemented with Root265‐derived PSs. *n* = 73 (Vehicle) and *n* = 61 (PSs) for day 2 adult worms, *n* = 116 (Vehicle) and *n* = 111 (PSs) for day 9 adult worms. ****p < 0.0001, *p < 0.01, ns denotes *p* > 0.05 via unpaired two‐tailed Student's *t* test. Error bars represent SEM. Source data for statistics are provided.

Breaking down liquid‐cultured Root265 collections into organic and aqueous phases along with cell debris (Figure S4c), we performed lifespan assays involving aqueous extracts mixed with live OP50 during day 1 to 5 of adulthood. Strikingly, supplementation of aqueous extracts alone led to lifespan extension (Figure 4a), suggesting the direct contribution of Root265‐derived components to the delay of host aging.

The cell envelope of mycobacteria harbors various aqueous‐soluble constituents, encompassing low molecular size lipoglycans (LAM and LM) and high molecular size polysaccharides (PSs) like glucan, mannan, arabinomannan, and arabinogalactan (Angala et al., 2018). SDS‐PAGE analysis revealed bands around 20KD and 30KD, along with smeared higher molecular size bands (>50KD), indicative of LAM, LM, and PSs within crude aqueous extracts (Figure S4d). Subsequently, separating original aqueous extracts using 50KD molecular sieves revealed that supplementation of extracts with >50KD molecular weight significantly extended the lifespan of *C. elegans* during day 1 to 5 of adulthood. However, LAM and LM mixtures did not produce similar effects (Figure S4e). Validating these findings, we extracted and purified PSs, demonstrating that their dietary supplementation during the same period was sufficient to extend the lifespan of *C. elegans* (Figure 4b,c) without significantly affecting the animals' pumping rate (Figure S7b). Additionally, PSs from *M. smegmatis* and *M. litorale* also extended the lifespan of *C. elegans* when supplemented during adulthood (day 1 to day 5) (Figure S4f,g). In contrast, components from the same extraction phase of *E. coli* OP50 did not prolong the lifespan (Figure S4h).

Considering the transcription factor DAF‐16/FOXO's known activation and responsibility in lifespan extension via various natural polysaccharides supplementation (Guo et al., 2022; Wang et al., 2019; Zhang et al., 2019), we examined whether DAF‐16 plays a role in the lifespan extension induced by Root265‐produced PSs. As demonstrated (Figure 4d), the *daf‐16*(*mgDf50*) mutant completely suppressed the lifespan‐promoting effects of Root265‐derived PSs. This suggests the requirement of the transcription factor DAF‐16 for the longevity effect of Root265‐derived PSs.

To test whether the lifespan‐promoting impact of Root265‐derived PSs correlated with the mitigation of age‐related physiological deterioration, we assessed motility, intestinal permeability, and BAS‐1::GFP levels in young and aged worms. Impressively, PSs maintained motility and intestinal barrier function in aged *C. elegans*, which typically decayed in animals fed OP50 (Figure 4e,f). However, PSs didn't rescue the age‐associated decline in BAS‐1 levels. In summary, Root265‐derived PSs emerge as key functional components responsible for extending host longevity in a *daf‐16*‐dependent manner, while also delaying late‐age onset issues in muscle and intestinal function.

### 2.5 *Mycobacterium* sp. Root265‐derived arabinogalactan peptidoglycan (AGP) extends lifespan and mitigates age‐related physiological decline

**FIGURE 5:**
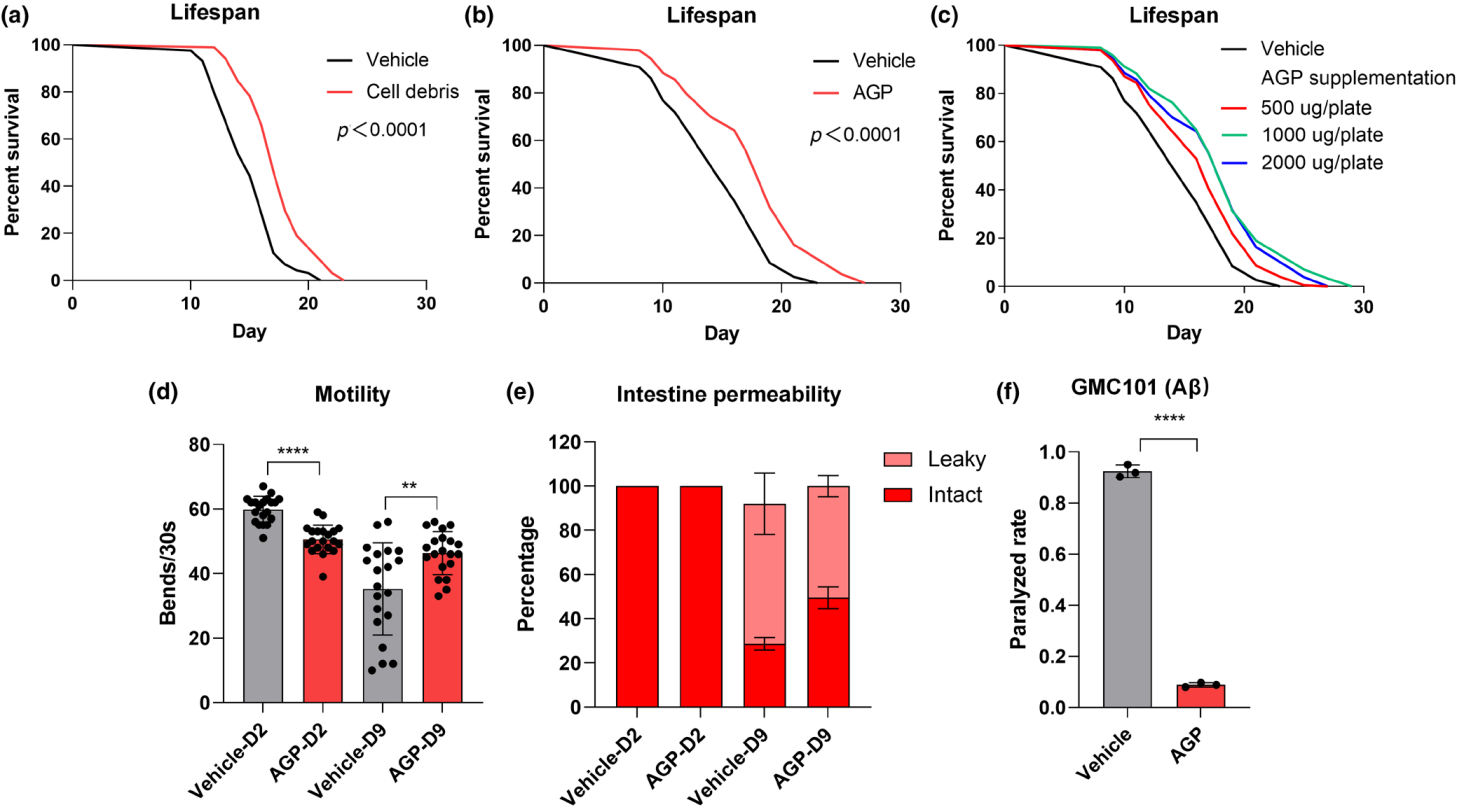
*Mycobacterium* sp. Root265‐derived AGP promotes the lifespan and alleviates Aβ‐induced toxicity in *C. elegans*. (a, b) Lifespan analysis of WT animals supplemented with cell debris (a) and AGP (b) extracted from Root265 during adulthood. (c) Lifespan analysis of WT animals supplemented with different concentrations of Root265‐derived AGP during adulthood. (d) Motility analysis of animals supplemented with Root265‐derived AGP on day 2 and day 9 of adulthood. *n* = 20 worms. (e) The percentage of animals with intact and leaky intestines on day 2 and day 9 of adulthood supplemented with Root265‐derived AGP. *n* = 88 (Vehicle) and *n* = 83 (AGP) for day 2 adult worms, *n* = 88 (Vehicle) and *n* = 99 (AGP) for day 9 adult worms. (f) Paralyzed rate of GMC101 animals supplemented with Root265‐derived AGP. *n* = 3 independent experiments. ****p < 0.0001, **p < 0.01, ns denotes *p* > 0.05 via unpaired two‐tailed Student's *t* test. Error bars represent SEM. Source data for statistics are provided.

Upon evaluating the impact of Root265's cell debris on *C. elegans* longevity, we noted a lifespan extension with its supplementation (Figure 5a). This cell debris primarily constitutes the cell wall core and peptidoglycan, structured with a repeating unit of N‐acetylglucosamine or N‐glycolylglucosamine linked with muramic acid and covalently attached to mycolyl‐arabinogalactan (mAG) (Alderwick et al., 2015).

Using biochemical separation and extraction, we successfully obtained purified AGP (arabinogalactan peptidoglycan), free of mycolic acid. As expected, AGP supplementation extended the lifespan of *C. elegans* (Figure 5b,c) without affecting the pumping rate of worms (Figure S7b). Interestingly, peptidoglycan (PG) derived from *E. coli* OP50 also promoted host longevity (Figure S5a), though its life‐prolonging effect was weaker compared to Root265‐derived AGP. This suggests that peptidoglycan, a polymer of linear glycan strands cross‐linked by short peptides, contributes to lifespan extension independently of its glycosylated modifications.

Remarkably, we observed that the *daf‐16*(*mgDf50*) mutant and *skn‐1*(*zj15*) mutant still exhibited substantial lifespan extension upon AGP supplementation. This suggests that AGP promotes host longevity through a *daf‐16*‐independent and *skn‐1*‐independent mechanism (Figure S5b,c). Additionally, simultaneous supplementation of PSs and AGP resulted in additive effects on lifespan extension, highlighting the lifespan‐enhancing roles of these two distinct components (Figure S5d).

To comprehend the influence of Root265‐derived AGP on age‐associated physiological deterioration, we assessed motility, intestinal permeability, and BAS‐1 protein levels in both young and aged animals. AGP supplementation effectively preserved motility and intestinal barrier integrity in aged animals (Figure 5d,e). Furthermore, when *C. elegans* models of Alzheimer's disease (AD) were administered AGP, the paralysis symptoms were alleviated, in line with the protective effects observed with Root265. However, AGP did not mitigate the age‐related decline in BAS‐1 protein levels, a marker of neuronal aging (data not shown).

To conclude, Root265‐derived AGP effectively counteracts aging and aging‐related deterioration in the muscle and intestine, offering promise in alleviating paralysis symptoms associated with AD and demonstrating its role in promoting healthy aging in *C. elegans*.

### 2.6 *Mycobacterium* sp. Root265‐derived polar lipids mitigate age‐related neuronal BAS‐1 decline

**FIGURE 6:**
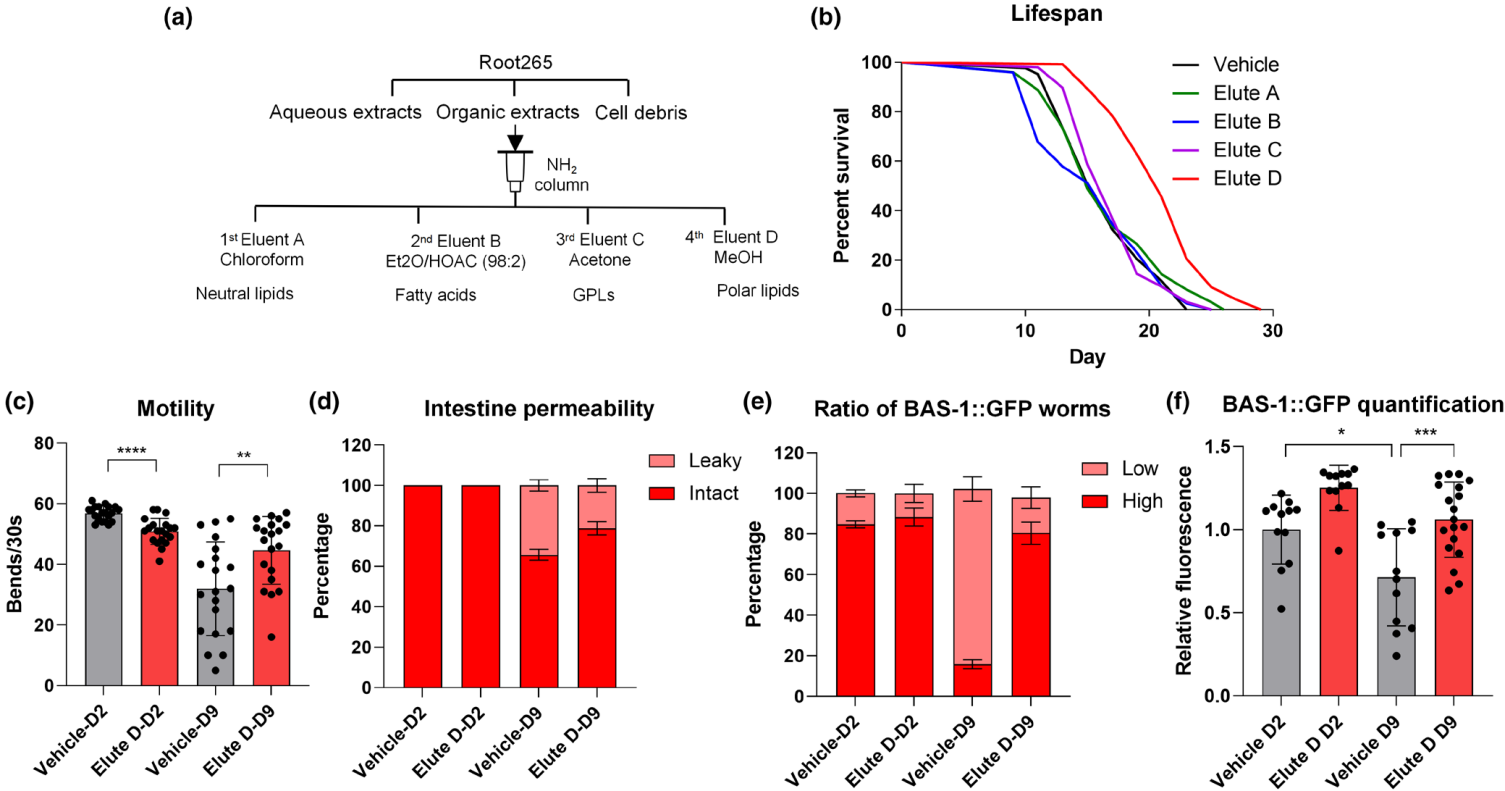
*Mycobacterium* sp. Root265‐derived polar lipids extend the lifespan and alleviate age‐related deterioration in neurons. (a) Schematic of fractionation of lipid classes using NH_2_ columns. (b) Lifespan analysis of WT animals supplemented with different organic fractions separated by SPE during adulthood. (c) Motility analysis of animals supplemented with Root265‐derived polar lipids (Elute D) at day 2 and day 9 of adulthood. *n* = 20 worms. (d) Percentage of animals with intact and leaky intestines on day 2 and day 9 of adulthood supplemented with Root265‐derived polar lipids (Elute D). *n* = 61 (Vehicle) and *n* = 74 (Elute D) for day 2 adult worms, *n* = 85 (Vehicle) and *n* = 86 (Elute D) for day 9 adult worms. (e) Percentage of animals supplemented with Root265‐derived polar lipids (Elute D) with high and low BAS‐1 levels. *n* = 64 (Vehicle) and *n* = 65 (Elute D) for day 2 adult worms, *n* = 62 (Vehicle) and *n* = 64 (Elute D) for day 9 adult worms. (f) Quantitative analysis of BAS‐1 levels performed by measuring GFP fluorescence intensity in the soma of NSM neurons. *n* = 12 (Vehicle) and *n* = 12 (Elute D) for day 2 adult worms, *n* = 12 (Vehicle) and *n* = 19 (Elute D) for day 9 adult worms. ****p < 0.0001, ***p < 0.001, **p < 0.01, *p < 0.05, ns denotes *p* > 0.05 via unpaired two‐tailed Student's *t* test. Error bars represent SEM. Source data for statistics are provided.

Mycobacteria are known for carrying an array of distinctive lipids (Jankute et al., 2015). Nonetheless, supplementation of total organic extracts (from day 1 to day 5) yielded no discernible effects on lifespan extension (Figure S6a). To further investigate the effective lipid components, total organic crude extracts underwent fractionation via solid‐phase extraction with different eluates based on polarity (Figure 6a) (Ruiz‐Gutiérrez & Pérez‐Camino, 2000). Intriguingly, only supplementation with the fourth elution, which primarily contained polar lipids, resulted in a significant extension of *C. elegans* lifespan (Figure 6b), without notable differences in the pumping rate compared to the control (Figure S7c). Moreover, polar lipid fractions isolated from the lifespan‐promoting *M. smegmatis* and *M. litorale* also positively influence lifespan (Figure S6b,c), whereas polar lipids from *E. coli* OP50 had no effect on longevity (Figure S6d).

Furthermore, we observed a remarkable lifespan extension in *daf‐16*(*mgDf50*) with polar lipids supplementation, suggesting that its lifespan‐promoting effect is independent of DAF‐16 (Figure S6e). Additionally, we found that simultaneous supplementation with three different pro‐longevity molecules (PSs, AGP, and polar lipids) resulted in a longer lifespan than any single molecule alone, confirming their distinct mechanisms for promoting longevity (Figure S6f).

To study the impact of polar lipid components on age‐onset physiologies, we assessed motility, intestinal permeability, and neuronal BAS‐1 levels in both young (day 2) and aged (day 9) *C. elegans*. The supplementation of polar lipid components not only restored the declined BAS‐1 levels in aged worms but also preserved better motility (Figure 6c–f). In summary, Root265‐derived polar lipids appear to not only extend host longevity but also delay age‐onset neuronal dysfunction.

## 3 DISCUSSION

The causal connection between microbial‐derived molecules and various aspects of host health and aging underscores the importance of investigating commensal microbial‐derived products and understanding their underlying mechanisms in the pursuit of aging‐delaying treatments (Cai et al., 2022). However, exploring these dynamics in mammalian model organisms is challenging due to complex microbiota composition and prolonged lifespans. The use of *C. elegans* in this research provides a simplified yet powerful platform to dissect the contribution of specific bacterial derivatives to host longevity and health. Its short lifespan and well‐defined genetics allow for rapid and precise investigations that would be challenging in more complex mammalian models. The findings presented here shed light on the multifaceted mechanisms underlying the effects of these microbial‐derived molecules on aging, including the involvement of key pathways such as DAF‐16, SKN‐1 and others.

The discovery that dead bacteria exert similar effects as live bacteria in extending lifespan is striking and aligns with previous findings involving heat‐killed *Lactobacillus paracasei* and *Bifidobacterium longum*, where cell components were shown to be the key drivers of lifespan extension rather than the localization or metabolites secreted by live bacteria (Wang et al., 2020; Zhao et al., 2017). This observation implies that specific bacterial cell components are responsible for the observed lifespan extension. In our study, we were able to identify three distinct lifespan‐promoting molecules: water‐soluble polysaccharides (PSs), water‐insoluble arabinogalactan peptidoglycan (AGP), and organic polar lipids. Moreover, we found that Root265‐derived PSs and AGP extend lifespan through distinguished pathways and exerted synergistic effects on host longevity. Furthermore, the AGP and the polar lipid components from Root265 were discovered to maintain homeostasis and mitigate age‐related decline in a neuronal marker, highlighting their potential to preserve neurosensory functions and potentially alleviate symptoms associated with neurodegenerative diseases such as Alzheimer's. The context‐specific impacts of bacterial components on host health and aging emphasize the need to isolate and study individual bacterial‐derived molecules. This approach not only deepens our understanding of the underlying mechanisms but also holds promise for interventions aimed at promoting healthy aging.

Natural macromolecules have been recognized for their remarkable bioactivities, such as immunomodulation, anti‐cancer properties, antioxidant effects, and cholesterol‐lowering capabilities, rendering them promising therapeutic alternatives (Griffin et al., 2021; Wheeler et al., 2014; Yu et al., 2018). Various natural polysaccharides have gained attention for their potential to extend lifespan (Wang et al., 2019). Notably, different types of polysaccharides, characterized by variations in structure and monosaccharide composition, have been associated with distinct longevity mechanisms, such as oxidative stress response, insulin/insulin‐like growth factor signaling (IIS) pathways, and Wnt/β‐catenin pathways (Guo et al., 2022; Lin et al., 2020). Mycobacteria Root265‐derived PSs extend lifespan through transcription factor FOXO/DAF‐16 independent of IIS, implying a novel longevity‐regulating pattern remains to be further identified.

The interplay between alterations in the gut microbiome and neurodegenerative diseases' causes or consequences remains enigmatic. Recent *C. elegans* research demonstrated that the probiotic *B. subtilis* shielded against a‐synuclein aggregation and amyloid‐β (Aβ)‐induced behavioral changes through live bacteria‐produced biofilm and stable bacterial components, respectively (Cogliati et al., 2020; Goya et al., 2019). Our study identified Root265‐derived AGP (glycosylated peptidoglycan), which specifically upholds protein homeostasis in the AD model. Bacterial peptidoglycan and its hydrolyzed derivatives serve as critical signaling molecules that modulate various host physiological processes, including immune responses, autophagy, apoptosis, and neuronal activities (Dziarski, 2004; Irazoki et al., 2019). In the case of the invertebrate model organism *C. elegans*, bacterial peptidoglycan derivatives, produced through extracellular and intracellular digestion (Bastos et al., 2021), function as potential molecular signals to modulate host longevity and homeostasis. Nevertheless, there is much to uncover in terms of host‐specific and peptidoglycan‐specific factors that contribute to these complex interactions. Future research holds the promise of shedding more light on these intricate mechanisms.

Moreover, short‐term interventions with Root265‐derived molecules during early adulthood yielded lasting anti‐aging effects, with earlier intervention proving more effective than late‐stage interventions. These findings propose a strategy for slowing the aging process through early‐life short‐term interventions, which may minimize side effects and improve feasibility (Juricic et al., 2022). Altogether, our study discovered novel anti‐aging bioactive compounds from a species of soil bacteria, which extend host longevity and counteract age‐related decay through diverse mechanisms, unveiling intricate bacterial‐host interplays. Further exploration is needed to understand these mechanisms and extend their potential for developing treatments targeting age‐related diseases in humans.

## 4 MATERIALS AND METHODS

### 4.1 *Caenorhabditis elegans* strains and maintenance

Nematodes *C. elegans* were maintained on nematode growth medium (NGM) plates seeded with *Escherichia coli* OP50 at 20°C unless otherwise noted. GMC101 (*dvIs100* [*unc‐54p*::*A‐beta‐1‐42*::*unc‐54* 3'‐UTR *+ mtl‐2p*::GFP]), CF512 (*rrf‐3*(*b26*); *fem‐1*(*hc17*)), Strains CL2166 (dvIs19 [(pAF15)*gst‐4p*::GFP::NLS]) and N2 wild‐type strains were obtained from the Caenorhabditis Genetics Center. Strains *Pbas‐1::bas‐1::gfp* were kindly gifted by Dr. Shiqing Cai's lab, at the University of Chinese Academy of Sciences, Shanghai, China. Strains MQD54 (*hqIs9* [*daf‐16p*::DAF‐16::6xHis::GFP, pRF4]) were gifted from Dr. Mengqiu Dong's lab at the National Institute of Biological Sciences, Beijing, China (Li et al., 2019).

### 4.2 Bacteria resource and growth

*Escherichia coli* OP50 was used as control bacteria in this study. *Arabidopsis* root‐associated bacterial isolate collection was kindly provided by Dr. Yang Bai's Lab at the Chinese Academy of Sciences, Beijing, China (Bai et al., 2015). Strain *M. smegmatis* MC^2^ 155 was gratefully gifted from Dr. Cuimin Liu's Lab, at the Chinese Academy of Sciences, Beijing, China. Stain *M. phlei* (CGMCC 4.1180) and *M. litorale* (CGMCC 4.5724) were obtained from China General Microbiological Culture Collection Center, Beijing, China.

For bacterial culture in a liquid medium, the single colony was incubated in TSB (Trypticase Soy Broth) at 30°C until to logarithmic phase (OD600 = 0.8 to 1.0). The well‐incubated bacteria were collected by centrifugation at 4000 *g* for 15 min. The bacterial pellets were washed using TSB buffer two times and then re‐suspended in TSB to a 5‐fold concentration.

For lifespan or other assay analysis, 150 μL concentrated bacterial suspension was seeded onto each 6 cm NGM plate. For killed‐bacteria treatment, 100 μL 5‐fold concentration bacterial suspension was seeded onto the NGM plates with kanamycin (50 mg/L) to inactive bacteria. In addition, bacteria were killed at 95°C for 1 hour.

### 4.3 Bacteria growth curves analysis

Pick up a single colony of *Mycobacterium* sp. Root265 from the TSB agar plate and inoculate it into 10 mL TSB liquid medium at 30°C overnight. Inoculate 2 mL of the overnight grown culture in 150 mL TSB liquid medium at 30°C. Bacteria were grown in 24‐well plates with shaking at 30°C. OD600 was recorded until to stationary phase.

### 4.4 Brood size

To evaluate the brood size, synchronized L4 hermaphrodites were transferred to NGM plates with seeded *E. coli* OP50 or *Mycobacterium* sp. Root265 at 20°C, then kept animals grow onto fresh dishes until reproductive cessation. The total number of progenies was calculated per individual. Two independent biological repeats were performed with six replicates per bacteria feeding treatment.

### 4.5 Developmental rate assay

To determine the developmental speed of *C. elegans*, animals were synchronized and about 350 eggs were allowed to be placed onto NGM plates with *E. coli* OP50 or *Mycobacterium* sp. Root265. Then worms were kept at 20°C for 70 hours and the number of worms at each developmental stage was calculated.

### 4.6 Movement in liquid media

Day 1 of adult animals were transferred to plate seeded with *E. coli* OP50, *Mycobacterium* sp. Root265 or with various kinds of supplementations. On Day 2 and Day 9, adulthood worms were transferred into a drop of M9 liquid buffer. After 15 seconds of adaption, the number of body bends was recorded for 30 seconds. A body bend was defined as a change in the direction of bending in the middle of the body. 10 worms per group and two independent biological repeats.

### 4.7 Measuring pharyngeal pumping rate

Worms were examined on NGM agar plates using a Leica M165 FC dissecting microscope for 10 s, and the number of pharynx pumping was scored manually.

### 4.8 Intestine permeability assay

Intestine barrier integrity assay was performed as described (Gelino et al., 2016). Day 1 adult animals were transferred to plate seeded with *E. coli* OP50, *Mycobacterium* sp. Root265 or with various kinds of supplementations. On day 2 and Day 9, adult animals were removed from the NGM plates and suspended in liquid cultures of standard OP50 bacteria (grown overnight) mixed with blue food dye (5% wt/vol) for 3 h. Worms were washed by M9 buffer for 5 times and then mounted and imaged using a Zeiss Imager M2 microscope.

### 4.9 Microscopy and image analysis

For whole‐worm fluorescence images, 10 to 15 worms were anesthetized with 50 mM sodium azide, photographs were taken using a Leica M165 FC dissecting microscope.

For quantifying the nuclear localization of DAF‐16::GFP, the worms were picked onto glass slides with 2% agarose pads and immobilized with 50 mM sodium azide, and photographs were taken using the Zeiss Imager M2 microscope. This protocol was also used to obtain fluorescence images of BAS‐1::GFP. The fluorescence intensities were quantified using software ImageJ.

### 4.10 PSs, AGP and polar lipids extraction

PSs and AGP were extracted as described (Lanéelle et al., 2015; Shenderov et al., 2013). In brief, Root265 was grown in TSB medium at 30°C for 4 days. Bacterial cells were collected by centrifugation and then were lyophilized. Lyophilized Root265 cells were ground with chloroform/methanol/water (2:1:1), and were fractionated into three layers, including the aqueous phase, the organic phase, and cell debris. For PS extraction, the crude polysaccharides (PSs) were precipitated with 95% EtOH (1:4, v/v) at 4°C for 12 h, repeated again. The protein was removed with butanol/chloroform (1:4) (1:1, v/v) for 10 times until the protein layer cannot be seen. Then PSs were precipitated with 95% EtOH (1:4, v/v) at 4°C for 12 h. Extracted PSs heated at 65°C for 50 min for sterilization.

For AGP extraction, cell debris was sonicated for 40 min, and then centrifuged 800 rpm for 10 min to remove unbroken cells. The pellets containing cell walls were re‐suspended in PBS, containing DNase and RNase to remove DNA/RNA. To remove protein, cell debris was treated in 10% SDS 60°C for 2 h, and then washed with water and 80% acetone twice. The treated pellets were washed with chloroform/methanol (2:1), and the supernatant was discarded. To remove the mycolic acids, the pellets were suspended in 0.5% KOH in methanol and stirred at 37°C for 2 days. The mixture was centrifuged, and the pellets were washed twice with methanol and with chloroform/methanol (2:1).

SPE‐NH_2_ column was used for lipids separation. The column was preconditioned with 3 volumes of chloroform, then the organic solution was put onto the column. The first fraction containing neutral lipids was eluted by chloroform/isopropanol (2:1), the second fraction containing fatty acids was eluted by diethyl ether/acetic acid (98:2), the third fraction mainly containing GPLs (glycopeptidolipids) (Whang et al., 2017) was eluted by acetone, the last fraction containing polar lipids was eluted by methanol (Ruiz‐Gutiérrez & Pérez‐Camino, 2000).

### 4.11 SDS‐PAGE and staining

As described, water‐soluble extracts were separated by SDS‐PAGE analysis and then stained (Lanéelle et al., 2015).

### 4.12 Dietary supplementation

For dietary supplementation of PSs and AGP, OP50 was seeded on the NGM and incubated overnight at 30°C (50 μL/plate). PSs were dissolved in water, achieving a concentration of 12.5 mg/mL, and seeded on the OP50 lawn. Due to AGP being insoluble in water, its water suspension (50 mg/mL) was added to the OP50 lawn for AGP supplementation. For dietary supplementation of organic extracts, organic extracts were dissolved in DMSO seeded on the NGM plates and dried in a clean bench, and then OP50 was seeded and cultured overnight at 30°C. The same volume of ddH_2_O or DMSO was used as the vehicle control. Worms were exposed to various supplements from Day 1 to Day 5 of adulthood.

### 4.13 Bomb calorimetry

The bacterial caloric count was measured following the procedure described by Stuhr and Curran (2020). Bacteria were cultured in TSB liquid medium until the log phase, then harvested by centrifugation and washed twice with PBS buffer to remove impurities. Excess water was removed, and the samples were frozen at −80°C. Triplicate samples were then sent to Hangzhou Young Instruments Science & Technology for drying and bomb calorimetry analysis.

### 4.14 Food clearance assay

The food clearance assay was conducted in liquid medium (S‐complete medium) following the method of Gomez‐Amaro et al. (2015), using 1.5 mL tubes. Each tube contained 150 μL bacterial culture (OD_600nm_ = 0.8) and 50 μg/mL kanamycin to prevent bacterial growth and contamination. To block self‐fertilization in the nematodes, 0.12 mM 5‐fluoro‐2′‐deoxyuridine (FUDR) was added to the medium. One hundred day‐1 worms were seeded into each tube, and bacterial optical density (OD600) was measured every 24 hours for 3 days. All measurements were performed in six replicates. The food clearance rate was determined by tracking the reduction in bacterial optical density over time.

### 4.15 Lifespan analysis

Lifespan analysis was performed on NGM plates at 20°C as previously (Dillin et al., 2002). Worms were synchronized by egg bleaching and were grown on *E. coli* OP50 or other bacterial cultures. The animals were scored every second day from Day 1 adult stage. All lifespan data are available in Table S2. Prism 8 software was used for statistical analysis, and the log‐rank (Mantel‐Cox) method was used to determine the significance difference.

### 4.16 Phylogenetic tree

16S rRNA gene sequence alignments were performed using Clustal X 2.1. Based on this multiple sequence alignment, the phylogenetic trees were constructed using FastTree 2.1.11 with the maximum‐likelihood method and MEGA 11 with the Neighbor‐joining method.

### 4.17 Statistical analysis

All experiments were conducted independently at least two or three times, consistently yielding identical results. Appropriate statistical tests were employed for each figure, with data meeting the necessary assumptions for the described tests. Statistical parameters, including the exact sample size (*n*), descriptive statistics (mean ± SEM), and significance levels, are reported in the figures and their legends. Unpaired two‐tailed student's *t* tests were used to compare two normally distributed groups. Lifespan assays were analyzed using the Mantel‐Cox log‐rank test. All original data are available in Table S3.

## AUTHOR CONTRIBUTIONS

Y.T. and L.L. conceived the study and designed the experiments. L.L. and X.S. performed the bacterial culture, lifespan experiments, and physiological phenotype measurements. L.L. conducted bacterial component separations and extractions. X.S. performed the calorie content test and food clearance assay of bacteria. Y.B. isolated and maintained bacterial isolates collection. X.S. designed the graphical abstract. X.S. and L.L. prepared the original figures. Y.T. and L.L. wrote the manuscript.

## CONFLICT OF INTEREST STATEMENT

The authors declare no conflicts of interest.

## Supporting information


**Figure S1.** Phylogenetic trees of *Arabidopsis* root bacterial isolates and *Mycobacterium* species. (a) Phylogenetic tree of *Arabidopsis* root bacterial isolates based on 16S rRNA gene sequences comparisons with the maximum‐likelihood method, 1000 bootstraps were carried out. *n* = 105 isolates. (b) Neighbor‐joining phylogenetic dendrogram of strain Root265 and related *Mycobacterium* species based on 16S rRNA gene sequences comparisons. The numbers indicate the bootstrap confidence values obtained for each node after 1000 replications.

**Figure S2.** *Mycobacterium* spp. extend the lifespan in wild type *C. elegans*. (a–c) Lifespan analysis of WT animals fed with three isolates of *Pseudomonas* spp. Root71 (a), Root9 (b), Root562 (c). (d, e) Lifespan analysis of WT animals fed with *M. smegmatis* MC2155, *M. litorale* CGMCC4.5724 (d) and *M. phlei* CGMCC4.1180 (e). (f) Growth curve of Root265 at 30°C. (g) 21 out of 119 bacterial isolates exhibit increased 14th survival rate in CF512 animals compared to OP50, 13 out of 21 isolates belonging to 8 genera extend lifespan significantly in WT animals.

**Figure S3.** IIS signaling pathway is not involved in *Mycobacterium* sp. Root265‐induced longevity. (a, b) Lifespan analysis of animals fed with OP50 and Root265 in *daf‐2*(*e1370*) (a) and *akt‐1*(*mg144*) (b) background. (c) Fluorescence visualization of the *gst‐4p::gfp* reporter. Scale bar, 250 μm.

**Figure S4.** *Mycobacterium* spp. ‐derived PSs extend the lifespan of *C. elegans*. (a, b) Lifespan analysis of WT animals supplemented with kanamycin‐killed (a) and heat‐killed Root265 (b). (c) Schematic of Root265 fractionation. (d) SDS‐PAGE analysis of aqueous extracts. (e) Lifespan analysis of WT animals supplemented with aqueous extracts of different molecular weights during adulthood. (f, g) Lifespan analysis of WT animals supplemented with polysaccharides extracted from *M. litorale* (f) and *M. smegmatis* (g). (h) Lifespan analysis of WT animals supplemented with polysaccharides extracted from *E. coli* OP50 and Root265.

**Figure S5.** *Mycobacterium* sp. Root265‐derived AGP extends the lifespan in *daf‐16*‐independent manner. (a) Lifespan analysis of WT animals supplemented with peptidoglycan from OP50 and Root265‐derived AGP. (b) Lifespan analysis of WT and *daf‐16*(*mgDf50*) mutant animals supplemented with Root265‐derived AGP. (c) Lifespan analysis of WT and *skn‐1*(*zj15*) mutant animals supplemented with Root265‐derived AGP. (d) Lifespan analysis of WT animals supplemented with both Root265‐derived AGP and PSs simultaneously.

**Figure S6.** *Mycobacterium* spp.—derived polar lipids extend the lifespan of *C. elegans*. (a) Lifespan analysis of WT animals supplemented with organic extracts from Root265 during adulthood. (b–d) Lifespan analysis of WT animals supplemented with polar lipids (Elute D) extracted from *M. litorale* (b), *M. smegmatis* (c) and *E. coli* OP50 (d) during adulthood. (e) Lifespan analysis of WT and *daf‐16*(*mgDf50*) mutant animals supplemented with Root265‐derived polar lipids (Elute D). (f) Lifespan analysis of WT animals supplemented with three types of Root265‐derived PSs, AGP and polar lipids (Elute D) simultaneously.

**Figure S7.** *Mycobacterium* sp. Root265 and Root265‐derived molecules don’t affect food intake in worms. (a) Pumping rate of day 2 adult worms fed with OP50 and Root265. *n* = 10 worms. (b, c) Pumping rate of day 2 adult worms supplemented with PSs and AGP (b) and polar lipids (Elute D) (c). *n* = 10 worms. (d) Caloric content of OP50 and Root265. (e) The bacterial clearance rates for OP50 and Root265 were similar, as determined by measuring the optical density (OD_600_) of each bacterial culture from Day 1 to Day 3.****p < 0.0001, ***p < 0.001, **p < 0.01, *p < 0.05, ns denotes *p* > 0.05 via unpaired two‐tailed Student’s *t* test. Error bars represent SEM. Source data for statistics are provided.

Table S1.

**Table S2.** Source data of all lifespan assays.

**Table S3.** Source data of other physiological phenotype assays.

## Data Availability

All data needed to evaluate the conclusions in this paper are present in the paper or the supplementary materials.
