# Supplementary figures and images for: The soil Mycobacterium sp. promotes health and longevity through different bacteria‐derived molecules in Caenorhabditis elegans
Source: Aging Cell. 2024 Nov 19;24(3):e14416. doi: 10.1111/acel.14416 (PMC11896450; doi:10.1111/acel.14416)

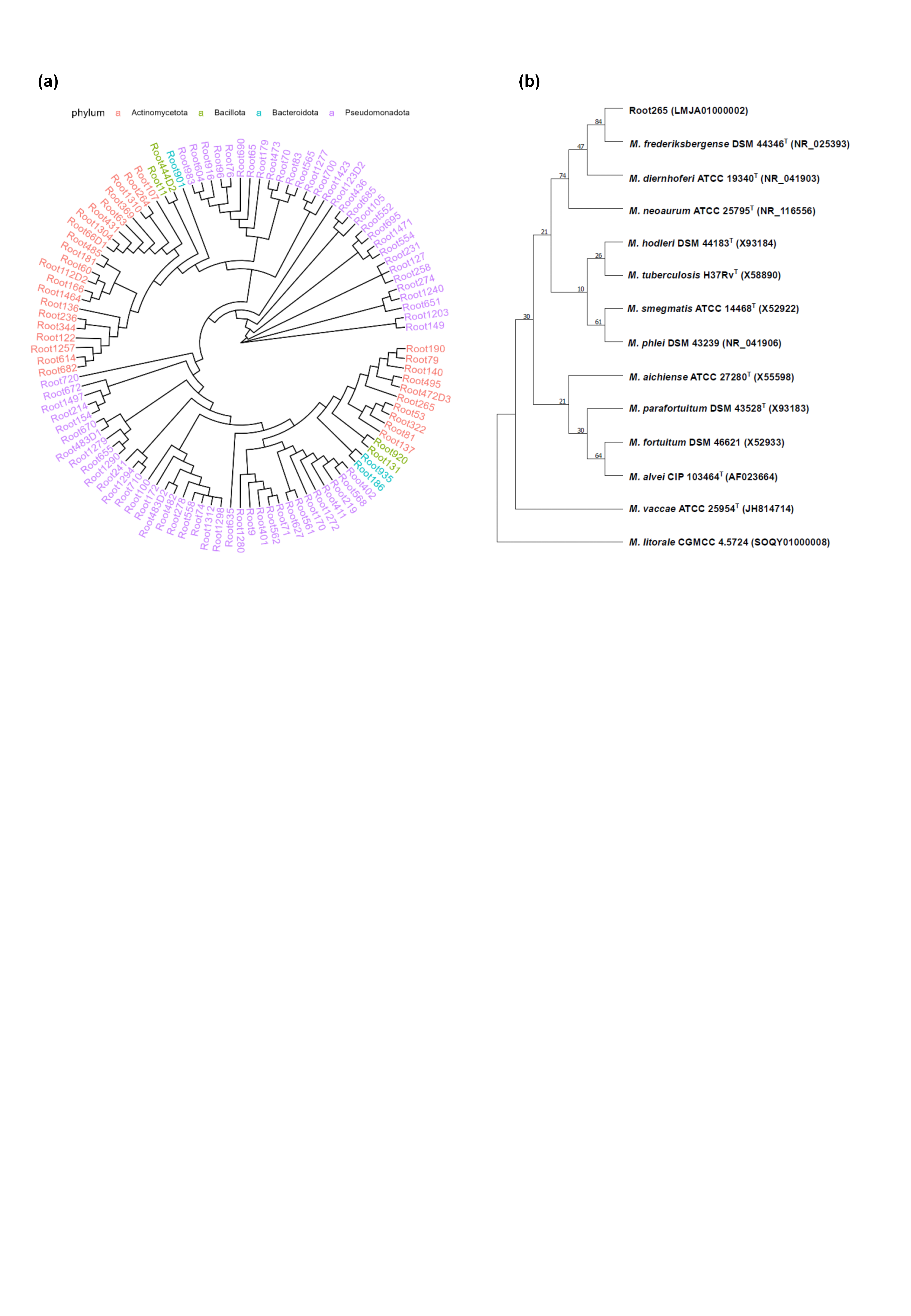

Supplement: Supplementary file 1 — Figure S1. Phylogenetic trees of Arabidopsis root bacterial isolates and Mycobacterium species. (a) Phylogenetic tree of Arabidopsis root bacterial isolates based on 16S rRNA gene sequences comparisons with the maximum‐likelihood method, 1000 bootstraps were carried out. n = 105 isolates. (b) Neighbor‐joining phylogenetic dendrogram of strain Root265 and related Mycobacterium species based on 16S rRNA gene sequences comparisons. The numbers indicate the bootstrap confidence values obtained for each node after 1000 replications. [file ACEL-24-e14416-s001.png]

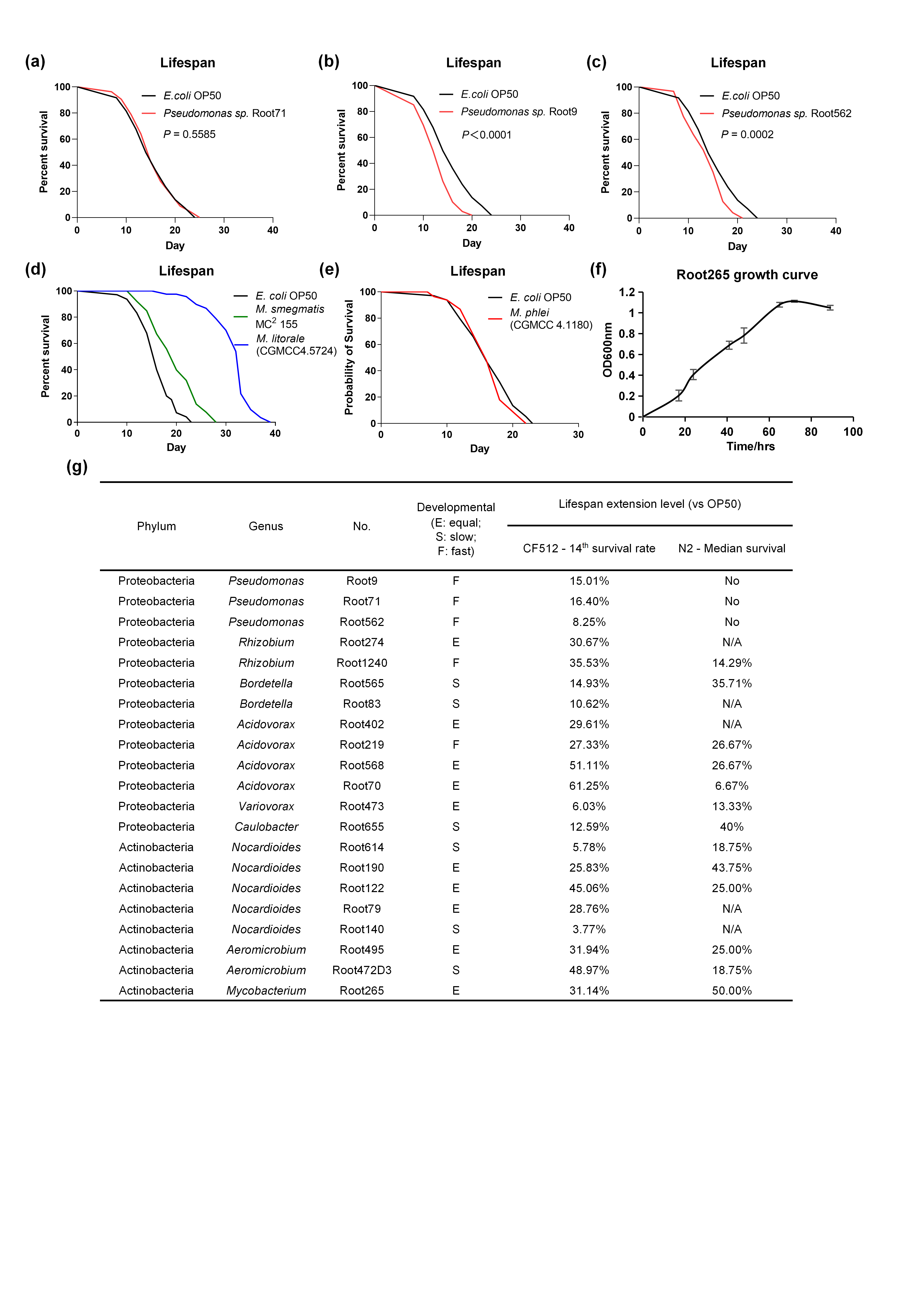

Supplement: Supplementary file 2 — Figure S2. Mycobacterium spp. extend the lifespan in wild type C. elegans. (a–c) Lifespan analysis of WT animals fed with three isolates of Pseudomonas spp. Root71 (a), Root9 (b), Root562 (c). (d, e) Lifespan analysis of WT animals fed with M. smegmatis MC2155, M. litorale CGMCC4.5724 (d) and M. phlei CGMCC4.1180 (e). (f) Growth curve of Root265 at 30°C. (g) 21 out of 119 bacterial isolates exhibit increased 14th survival rate in CF512 animals compared to OP50, 13 out of 21 isolates belonging to 8 genera extend lifespan significantly in WT animals. [file ACEL-24-e14416-s002.png]

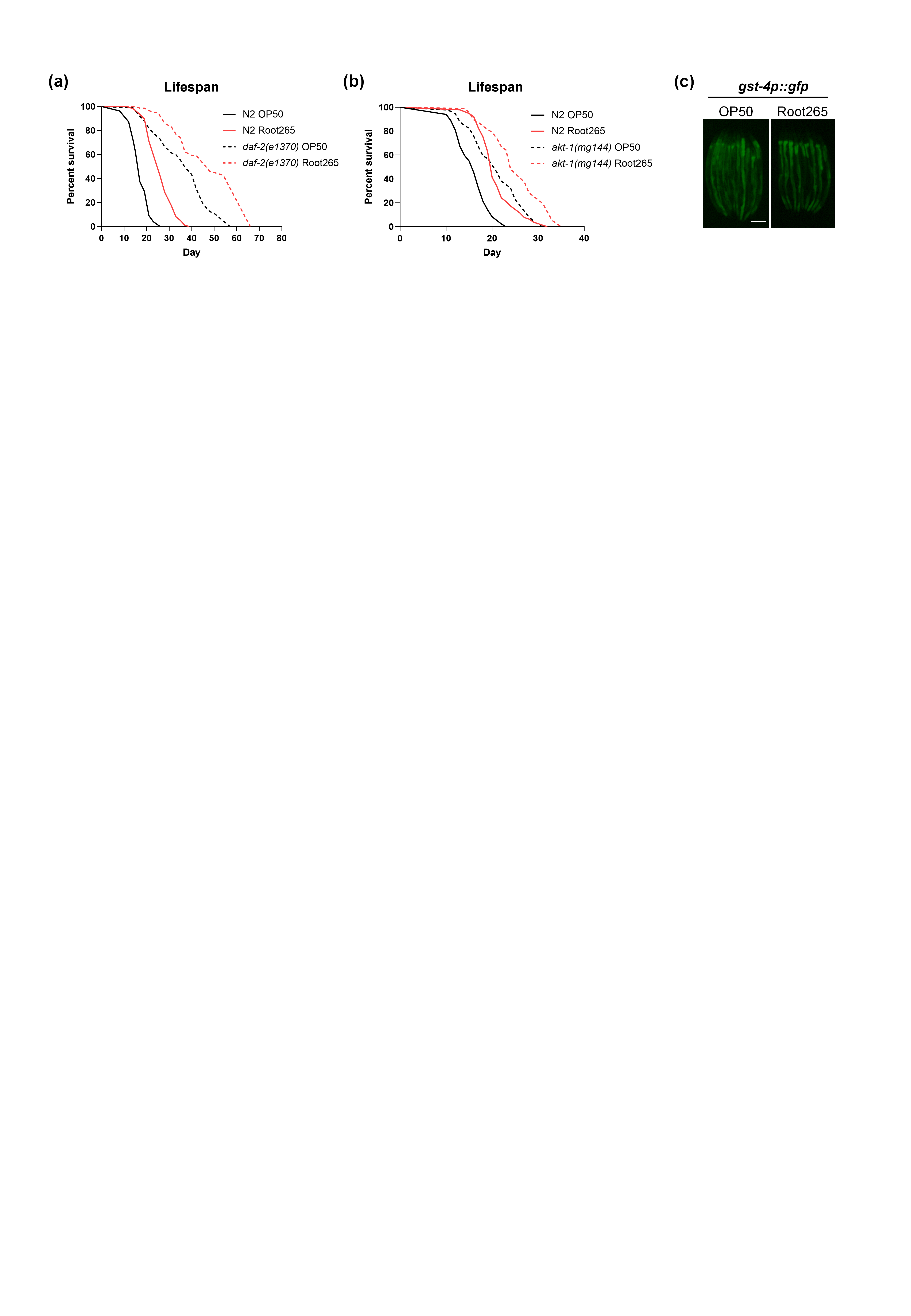

Supplement: Supplementary file 3 — Figure S3. IIS signaling pathway is not involved in Mycobacterium sp. Root265‐induced longevity. (a, b) Lifespan analysis of animals fed with OP50 and Root265 in daf‐2(e1370) (a) and akt‐1(mg144) (b) background. (c) Fluorescence visualization of the gst‐4p::gfp reporter. Scale bar, 250 μm. [file ACEL-24-e14416-s005.png]

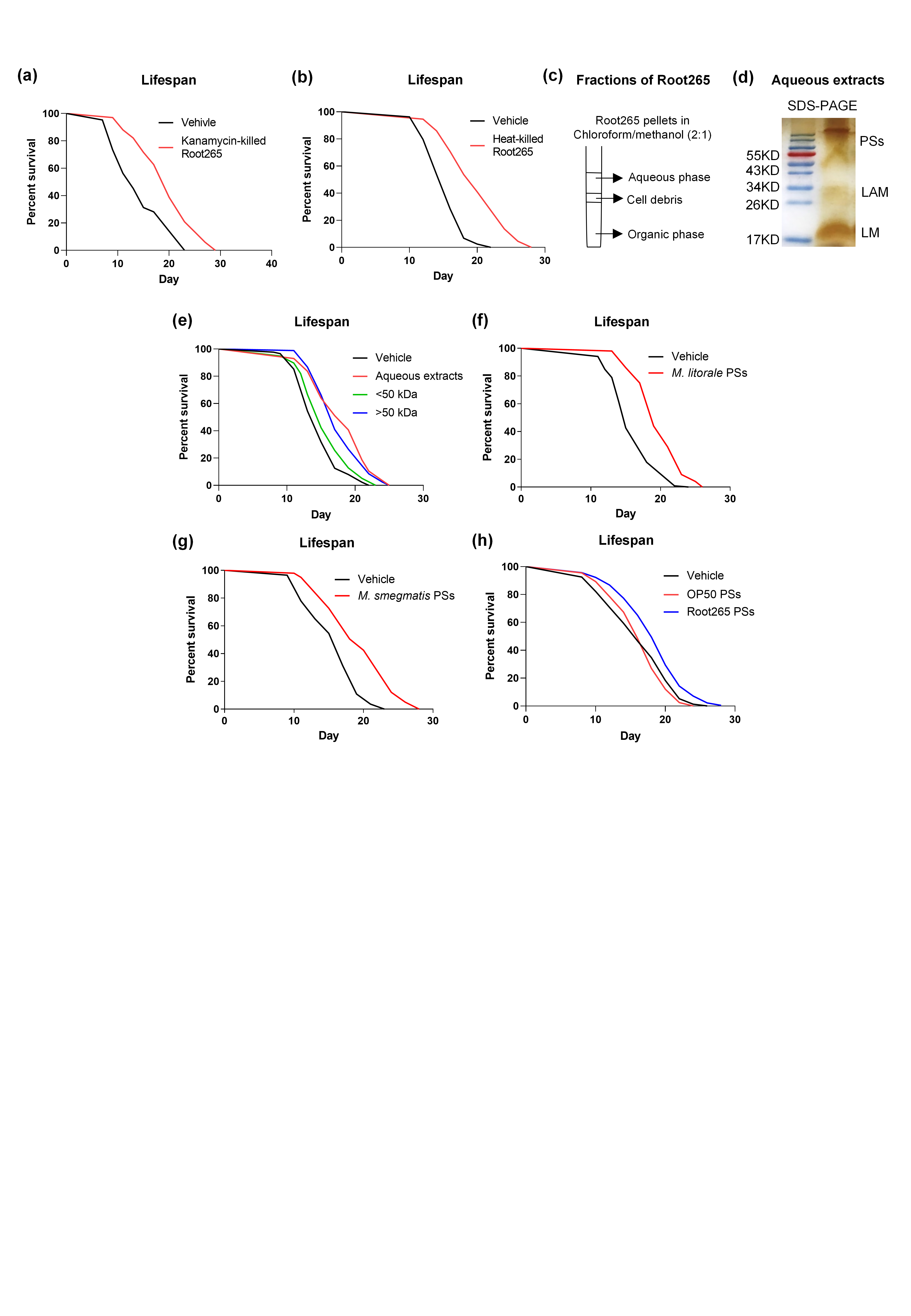

Supplement: Supplementary file 4 — Figure S4. Mycobacterium spp. ‐derived PSs extend the lifespan of C. elegans. (a, b) Lifespan analysis of WT animals supplemented with kanamycin‐killed (a) and heat‐killed Root265 (b). (c) Schematic of Root265 fractionation. (d) SDS‐PAGE analysis of aqueous extracts. (e) Lifespan analysis of WT animals supplemented with aqueous extracts of different molecular weights during adulthood. (f, g) Lifespan analysis of WT animals supplemented with polysaccharides extracted from M. litorale (f) and M. smegmatis (g). (h) Lifespan analysis of WT animals supplemented with polysaccharides extracted from E. coli OP50 and Root265. [file ACEL-24-e14416-s006.png]

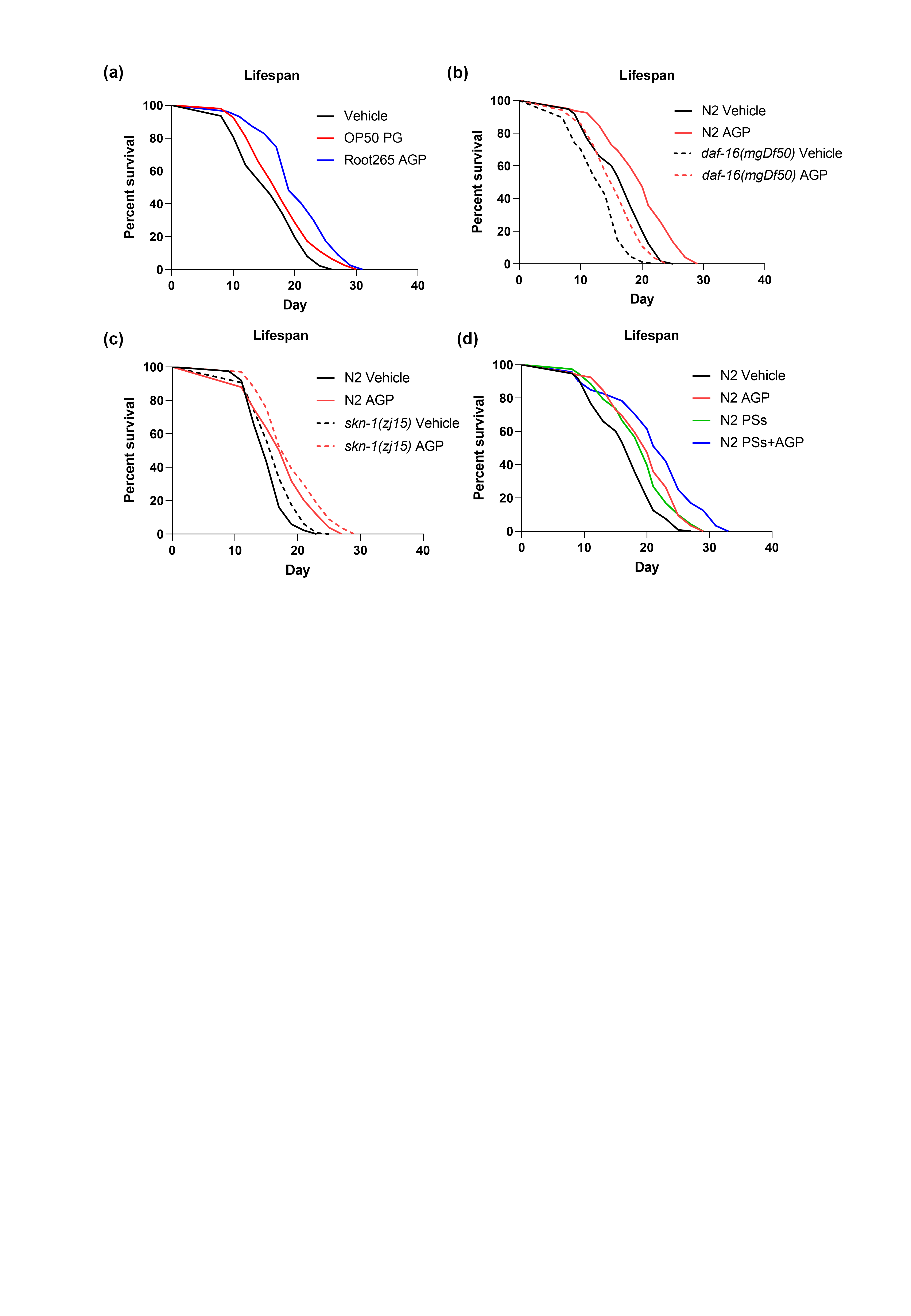

Supplement: Supplementary file 5 — Figure S5. Mycobacterium sp. Root265‐derived AGP extends the lifespan in daf‐16‐independent manner. (a) Lifespan analysis of WT animals supplemented with peptidoglycan from OP50 and Root265‐derived AGP. (b) Lifespan analysis of WT and daf‐16(mgDf50) mutant animals supplemented with Root265‐derived AGP. (c) Lifespan analysis of WT and skn‐1(zj15) mutant animals supplemented with Root265‐derived AGP. (d) Lifespan analysis of WT animals supplemented with both Root265‐derived AGP and PSs simultaneously. [file ACEL-24-e14416-s004.png]

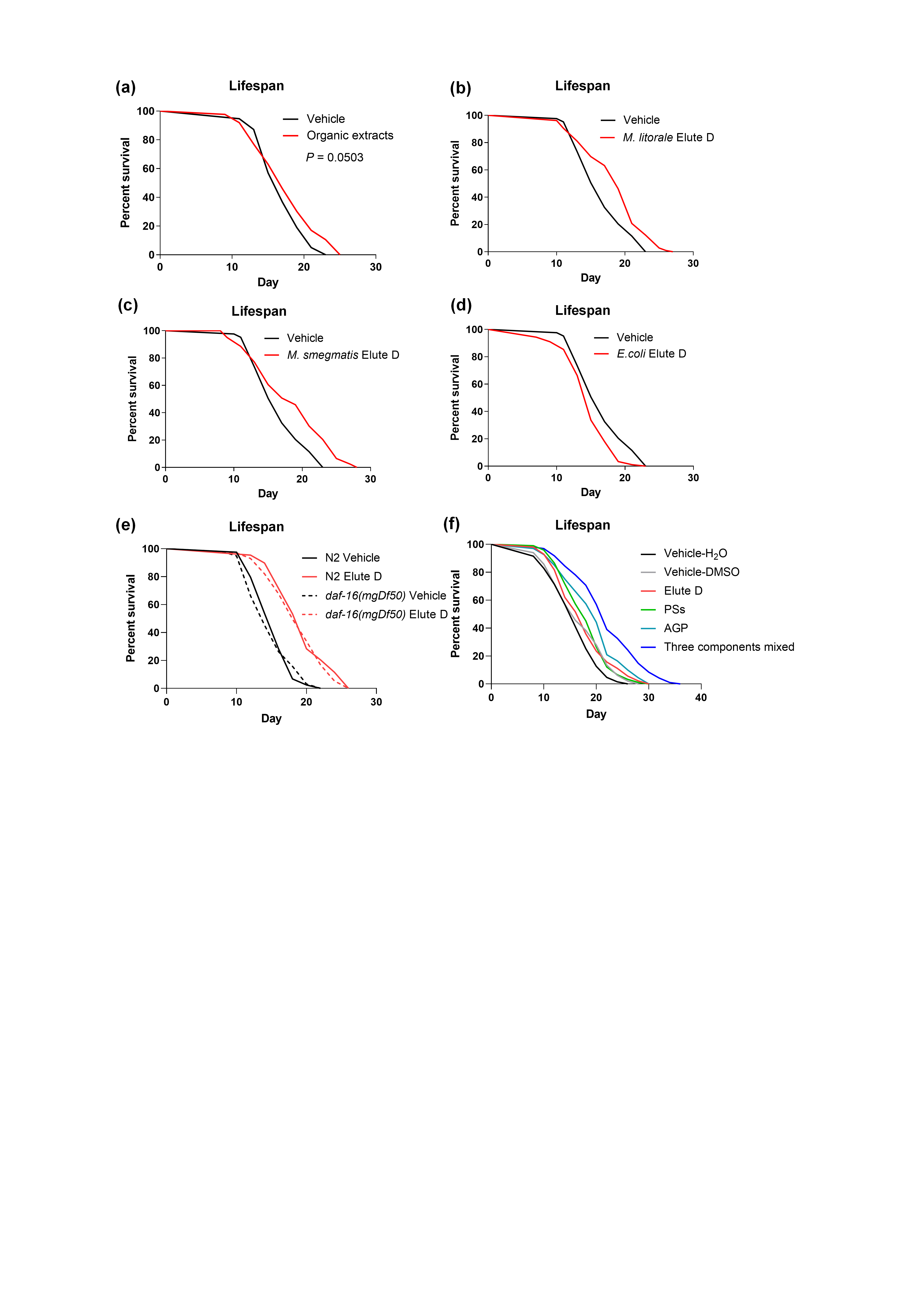

Supplement: Supplementary file 6 — Figure S6. Mycobacterium spp.—derived polar lipids extend the lifespan of C. elegans. (a) Lifespan analysis of WT animals supplemented with organic extracts from Root265 during adulthood. (b–d) Lifespan analysis of WT animals supplemented with polar lipids (Elute D) extracted from M. litorale (b), M. smegmatis (c) and E. coli OP50 (d) during adulthood. (e) Lifespan analysis of WT and daf‐16(mgDf50) mutant animals supplemented with Root265‐derived polar lipids (Elute D). (f) Lifespan analysis of WT animals supplemented with three types of Root265‐derived PSs, AGP and polar lipids (Elute D) simultaneously. [file ACEL-24-e14416-s003.png]

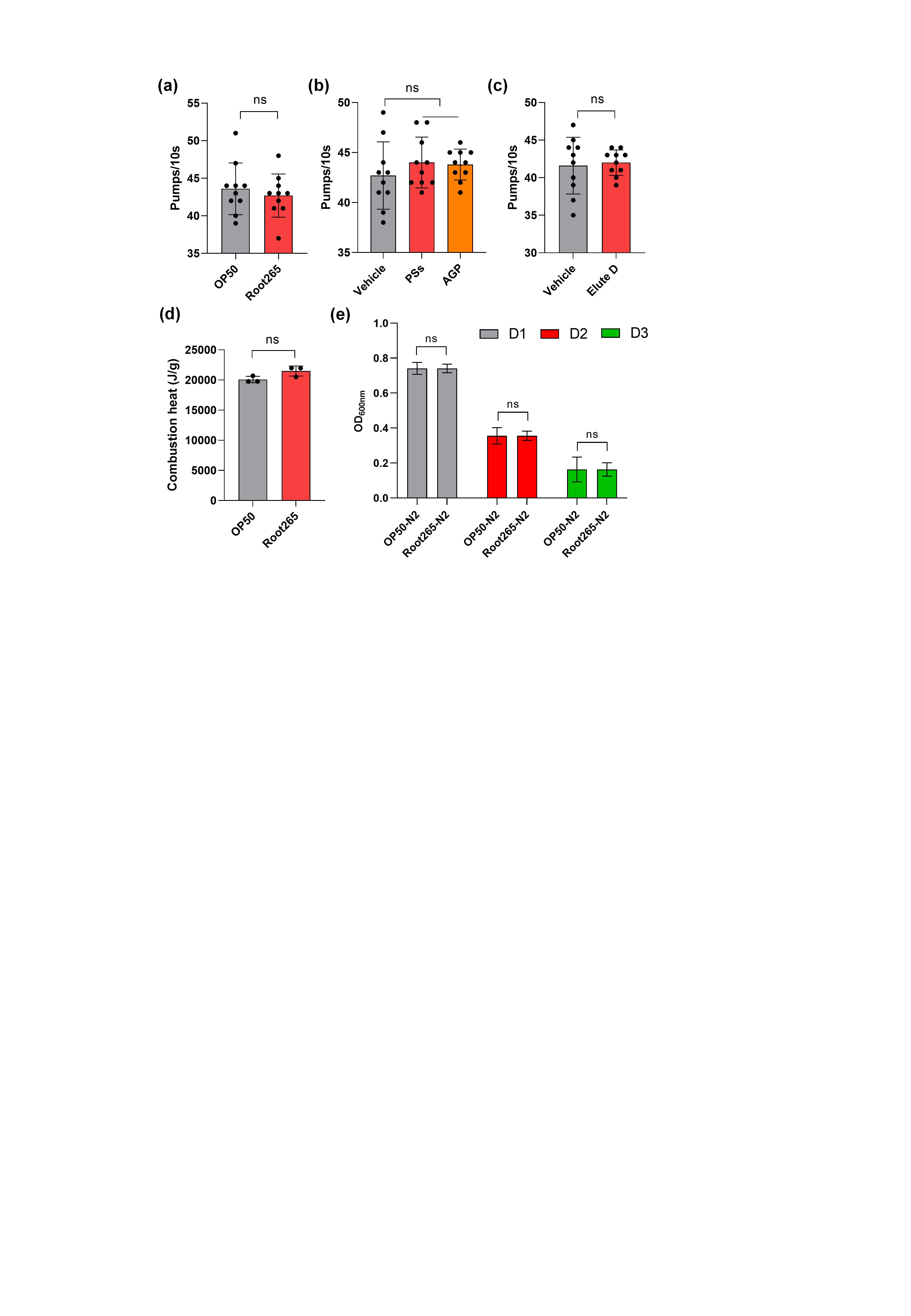

Supplement: Supplementary file 7 — Figure S7. Mycobacterium sp. Root265 and Root265‐derived molecules don’t affect food intake in worms. (a) Pumping rate of day 2 adult worms fed with OP50 and Root265. n = 10 worms. (b, c) Pumping rate of day 2 adult worms supplemented with PSs and AGP (b) and polar lipids (Elute D) (c). n = 10 worms. (d) Caloric content of OP50 and Root265. (e) The bacterial clearance rates for OP50 and Root265 were similar, as determined by measuring the optical density (OD600) of each bacterial culture from Day 1 to Day 3.****p < 0.0001, ***p < 0.001, **p < 0.01, *p < 0.05, ns denotes p > 0.05 via unpaired two‐tailed Student’s t test. Error bars represent SEM. Source data for statistics are provided. [file ACEL-24-e14416-s007.png]
